# Supplementary material for: Regulation of vascular endothelial integrity by mesenchymal stem cell extracellular vesicles after hemorrhagic shock and trauma
Source: J Transl Med. 2024 Jun 21;22:588. doi: 10.1186/s12967-024-05406-1 (PMC11191310; doi:10.1186/s12967-024-05406-1)

**Scanning Electron Microscopy (SEM) of MSC EVs**

The MSC EVs used in this study were previously characterized by flow cytometry, spectrophotometry, and nanoparticle tracking analysis by Barry et al.^12^ In addition, we used scanning electron microscopy (SEM) to image the MSC EVs used in this study. MSC EVs were fixed in 1% glutaraldehyde for 1min then ultracentrifuged for 2 hours at 68,000RPM at 2°C using a Optima^TM^ TLX Ultracentrifuge (Beckman Coulter, Inc., Brea, CA). At 5°C, the EV pellet was rinsed 3x with distilled water, post-fixed with 1% osmium tetroxide for 1 hour, then rinsed again 3x with distilled water. The bottom of the centrifuge tube containing the EV pellet was cut off, rinsed of debris, and placed in a freeze-drying fixture. After freeze-dry, approximately 10nm gold was sputter-coated on the sample. Flakes of the dried pellet were transferred to an adhesive carbon tape for SEM viewing in secondary electrode (SE) mode at 20KV accelerating voltage using a TESCAN VEGA 3 Scanning Electron Microscope (TESCAN, Brno, Czechia). An image of the EVs is depicted in Supplemental Figure 1.

**Supplemental Figure 1. MSC EV Scanning Electron Microscopy.** SEM image at 100KX magnification demonstrating an agglomeration of EV particles of the expected size.


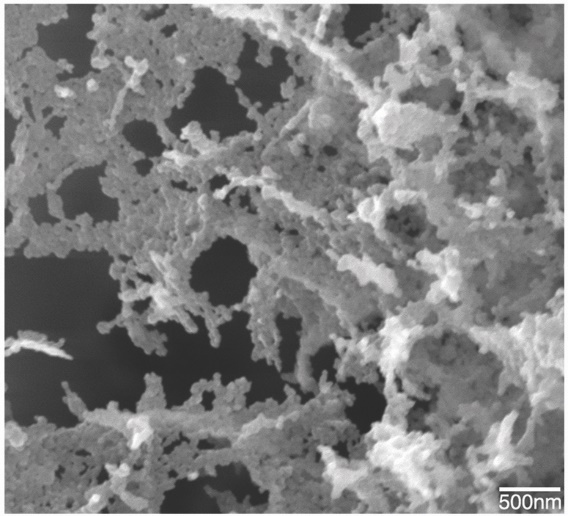

Supplement: Supplementary file 1 — Supplementary Material 1. [file 12967_2024_5406_MOESM1_ESM.docx]
